# JAK‐Inhibitoren und Veränderungen des Menstruationszyklus: Erkenntnisse aus einer Fallserie mit 16 Patientinnen

**DOI:** 10.1111/ddg.70173x

**Published:** 2026-09-08

**Authors:** Luciano Ibba, Luigi Gargiulo, Matteo Bianco, Sara Di Giulio, Ruggero Cascio Ingurgio, Angela Alfano, Carlo A. Vignoli, Mario Valenti, Paola Facheris, Chiara Perugini, Alessandra Narcisi, Antonio Costanzo

**Affiliations:** ^1^ Dermatology Unit IRCCS Humanitas Research Hospital Rozzano Milan Italy; ^2^ Department of Biomedical Sciences Humanitas University Pieve Emanuele Milan Italy

Sehr geehrte Herausgeber,

Januskinase (JAK)‐Inhibitoren haben sich als wichtige Behandlungsmethoden für verschiedene Hauterkrankungen, darunter atopische Dermatitis (AD) und Alopecia areata (AA), etabliert.^1^

Ihre Anwendung ist jedoch mit unerwünschten Ereignissen (*adverse events*, AEs),) verbunden, die die Fortsetzung der Therapie und die allgemeine Lebensqualität der Patienten beeinträchtigen können.^2^ Kürzlich wurde über einen Fall von Amenorrhö bei einer Patientin berichtet, die wegen AD mit Upadacitinib behandelt wurde.^3^ Der Mechanismus, der diesem AE zugrunde liegt, ist noch nicht vollständig geklärt. Leptin könnte eine entscheidende Rolle bei der Regulierung der Hypothalamus‐Hypophysen‐Ovar‐Achse (HPO) über den JAK2/STAT3‐Signalweg spielen. JAK‐Inhibitoren können die Leptin‐Signalübertragung verändern und dadurch die Sekretion des Gonadotropin‐freisetzenden Hormons (GnRH) und die Gonadotropin‐Freisetzung stören, was zu Menstruationsstörungen führt.^4^, ^5^

Baricitinib (2/4 mg) hemmt JAK1/2 und ist für mittelschwere bis schwere AD und schwere AA zugelassen.^1^ Abrocitinib (100/200 mg) hemmt selektiv JAK1 bei mittelschwerer bis schwerer AD. Upadacitinib, ein JAK1‐Inhibitor, ist in Dosierungen von 15 mg und 30 mg für mittelschwere bis schwere AD zugelassen, wobei In‐vitro‐Studien darauf hindeuten, dass 30 mg auch die JAK2‐Signalübertragung hemmen können.^1^, ^6^, ^7^ Die häufig mit JAK‐Inhibitoren verbundenen AEs sind in der Regel leicht bis mittelschwer und umfassen nicht‐infektiöse und infektiöse kutane Symptome sowie Laborwertabweichungen.^1^

Wir berichten über eine Fallserie mit 16 Patientinnen, bei denen während der Behandlung mit JAK‐Inhibitoren gegen AA oder AD Menstruationsstörungen auftraten. Wir schlossen Patientinnen aus, die bereits vor Beginn der JAK‐Inhibitor‐Therapie über unregelmäßige Menstruationszyklen berichtet hatten. Die Merkmale der Patientinnen zu Beginn der Studie wurden retrospektiv aus den Krankenakten des IRCCS Humanitas Research Hospital (Mailand, Italien) abgerufen. Die Patientinnen wurden bei jeder Kontrolle auf Zyklusveränderungen (einschließlich Amenorrhö, Hyper‐/Hypomenorrhö, Poly‐/Oligomenorrhö und Dysmenorrhö) untersucht.^8^ Kontinuierliche Variablen wurden als Mittelwert und Standardabweichung (SD) angegeben, während kategoriale Variablen als absolute Zahlen und Prozentsätze ausgedrückt wurden.

Eine Genehmigung durch die Ethikkommission war nicht erforderlich, da die Studienverfahren nicht von der guten klinischen Praxis abwichen. Die Studie wurde gemäß der Helsinki‐Erklärung von 1964 und ihren späteren Ergänzungen durchgeführt. Alle eingeschlossenen Patientinnen willigten schriftlich zur retrospektiven Analyse ihrer klinischen Daten ein.

Unsere Population umfasste 16 Patientinnen mit AA oder AD, die mit JAK‐Inhibitoren behandelt wurden (Tabelle 1). Das Durchschnittsalter zu Studienbeginn betrug 35 Jahre (SD 10), der durchschnittliche *Body‐Mass‐Index* (BMI) lag bei 20 (SD 2). Drei Patientinnen (19 %) nahmen bereits orale Kontrazeptiva ein. Neun Patientinnen (56 %) erhielten 4 mg Baricitinib wegen schwerer AA, während die anderen sieben (45 %) wegen mittelschwerer bis schwerer AD mit Upadacitinib behandelt wurden (5 Patientinnen 15 mg; 2 Patientinnen 30 mg). Die am häufigsten berichtete Veränderung des Menstruationszyklus war Amenorrhö (8 Patientinnen, 50 %), gefolgt von Polymenorrhö (6 Patientinnen, 38 %). Unsere Patientinnen entwickelten Menstruationsstörungen nach einer mittleren Zeit von 5 Monaten (SD 4) seit Beginn der Behandlung. Alle Patientinnen, die eine Amenorrhö entwickelten, setzten das Medikament ab, unterzogen sich einer gynäkologischen Untersuchung mit negativen Ergebnissen hinsichtlich organischer Veränderungen und bekamen ihren normalen Menstruationszyklus wieder.

**TABELLE 1:** Individuelle Patientendaten zu Veränderungen des Menstruationszyklus bei 16 Patientinnen, die wegen AD oder AA mit JAK‐Inhibitoren behandelt wurden.

Patientennummer	Alter	Gewicht (kg)	Größe (cm)	BMI	Begleiterkrankungen	Hauterkrankung	Orale Kontrazeptiva	JAK‐Hemmer	Monate	Menstruationsveränderungen	Medikament abgesetzt?	Aufhebung der Menstruationsveränderung?
1	42	60	162	23		AD	Nein	Upadacitinib 15 mg	17,8	Polymenorrhoe	Nein	
2	25	57	160	22		AD	Nein	Upadacitinib 15 mg	6,0	Amenorrhö	Ja	Ja
3	25	56	172	19		AD	Nein	Upadacitinib 30 mg	7,1	Polymenorrhoe	Nein	
4	47	52	160	20		AD	Ja	Upadacitinib 15 mg	2,5	Polymenorrhoe	Nein	
5	25	60	165	22		AD	Nein	Upadacitinib 15 mg	1,3	Amenorrhö	Ja	Ja
6	31	48	160	19		AD	Nein	Baricitinib 4 mg	0,8	Amenorrhö	Ja	Ja
7	23	50	160	20		AD	Nein	Upadacitinib 15 mg	1,0	Oligomenorrhoe	Nein	
8	47	49	154	21		AD	Nein	Upadacitinib 30 mg	2,4	Amenorrhö	Ja	Ja
9	38	50	168	18		AA	Nein	Baricitinib 4 mg	11,5	Polymenorrhoe	Nein	
10	53	55	165	20	Hypercholesterinämie	AA	Nein	Baricitinib 4 mg	3,9	Amenorrhö	Ja	Ja
11	31	47	158	19		AA	Nein	Baricitinib 4 mg	1,8	Amenorrhö	Ja	Ja
12	45	55	160	21		AA	Nein	Baricitinib 4 mg	6,2	Polymenorrhoe	Nein	
13	33	55	163	21	Hypercholesterinämie	AA	Ja	Baricitinib 4 mg	7,3	Amenorrhö	Ja	Ja
14	39	45	160	18	Hypercholesterinämie	AA	Nein	Baricitinib 4 mg	5,1	Oligomenorrhoe	Nein	
15	21	53	160	21		AA	Nein	Baricitinib 4 mg	5,9	Polymenorrhoe	Nein	
16	36	51	160	20	Thyreopathie	AA	Ja	Baricitinib 4 mg	1,4	Amenorrhö	Ja	Ja

*Abkürzungen*: BMI, *Body Mass Index*; JAK, Janus‐Kinase; AA, Alopecia areata; AD, atopische Dermatitis.

Die Definitionen für Veränderungen des Menstruationszyklus basierten auf den FIGO‐Kriterien: Amenorrhö wurde definiert als das Ausbleiben der Menstruationsblutung für ≥ 3 aufeinanderfolgende Monate; Polymenorrhö als eine Menstruationszykluslänge von < 21 Tagen; Oligomenorrhö als eine Menstruationszykluslänge von > 35 Tagen.^8^

Unsere Fallserie hebt einen möglichen Zusammenhang zwischen JAK‐Inhibitoren und Veränderungen des Menstruationszyklus bei Patientinnen hervor, die wegen AD oder AA behandelt wurden. Amenorrhö war die am häufigsten berichtete Veränderung, was auf eine mögliche Auswirkung auf die HPO‐Achse hindeutet. Die meisten Patientinnen (11; 69 %) erhielten eine Therapie, die auch auf die Signalübertragung von JAK2 wirkte (9 mit Baricitinib 4 mg und 2 mit Upadacitinib 30 mg). Die JAK2‐Signalübertragung ist an leptinvermittelten Effekten auf hypothalamischer Ebene beteiligt, und ihre Hemmung kann die GnRH‐Pulsatilität stören, was zu Menstruationsstörungen führt.^4^, ^5^ Eine aktuelle Studie hat gezeigt, dass die Unterdrückung dieses Signalwegs eine metabolische Dysregulation der Leptinaktivität induzieren kann, was zu signifikanter Gewichtszunahme bei Patientinnen führt, die mit Wirkstoffen behandelt werden, die die JAK2‐vermittelte Signalübertragung beeinträchtigen.^9^

Menstruationsstörungen traten im Durchschnitt 5 Monate nach Beginn der Behandlung mit dem JAK‐Inhibitor auf, was eine verzögerte Wirkung auf die Hormonregulation nahelegt. Bei acht Patientinnen klang die Amenorrhö nach Absetzen der JAK‐Inhibitoren von selbst ab, was auf eine reversible medikamentenbedingte Wirkung hindeutet.

Unsere Studie hat mehrere Einschränkungen, darunter ihr retrospektives Design und die relativ kleine Stichprobengröße. Darüber hinaus ermöglichten der retrospektive Charakter der Analyse und die Variabilität der von den Patientinnen angegebenen Daten keine Erstellung eines konsistenten und zuverlässigen Zeitverlaufs. Dennoch hatten alle Patientinnen bei der ersten Kontrolluntersuchung sechs Monate nach Absetzen des Medikaments wieder normale Menstruationszyklen. Obwohl Patientinnen mit vorbestehenden Menstruationsstörungen ausgeschlossen wurden, können unerkannte endokrine Störungen oder der Einfluss von Begleitmedikamenten nicht vollständig ausgeschlossen werden.

Zusammenfassend lässt sich sagen, dass JAK‐Inhibitoren, insbesondere solche, die die JAK1/2‐Signalübertragung blockieren, die HPO‐Achse stören und zu Veränderungen des Menstruationszyklus beitragen können. Ärzte sollten sich dieser potenziellen Nebenwirkungen bewusst sein und den Menstruationszyklus von Patientinnen, die diese Medikamente einnehmen, überwachen. Um diese Ergebnisse zu bestätigen, sind größere und längerfristige Studien mit Hormonprofilierung erforderlich.

## ETHIKERKLÄRUNG

Eine Genehmigung durch die Ethikkommission war nicht erforderlich, da die Studienverfahren nicht von der guten klinischen Praxis abwichen. Die Studie wurde gemäß der Helsinki‐Erklärung von 1964 und ihren späteren Ergänzungen durchgeführt. Alle in die Studie einbezogenen Patientinnen willigten schriftlich in die retrospektive Analyse ihrer klinischen Daten ein.

## INTERESSENKONFLIKT

L.I. war als Berater für Almirall tätig. L.G. war als Berater und/oder Referent tätig und hat in Beiräten für AbbVie, Almirall, Eli Lilly, Pfizer, Sanofi und UCB mitgewirkt. M.V. war als Berater und/oder Referent für Sanofi, LEO Pharma, Eli Lilly, Novartis, Janssen, AbbVie, Boehringer Ingelheim, Almirall, UCB und Difa Cooper tätig. P.F. war als Berater für Eli Lilly und als Referent für UCB, Pfizer und AbbVie tätig. A.N. war Mitglied in Beiräten und erhielt Honorare für Vorträge und Forschungsstipendien von Almirall, AbbVie, LEO Pharma, Celgene, Eli Lilly, Janssen, Novartis, Sanofi Genzyme, Amgen und Boehringer Ingelheim. A.C. war als Beiratsmitglied und Berater tätig und hat Honorare und Vortragshonorare erhalten oder an klinischen Studien für AbbVie, Almirall, Biogen, LEO Pharma, Eli Lilly, Janssen, Novartis, Pfizer, Sanofi Genzyme und UCB teilgenommen. Alle anderen Autoren erklären, dass keine Interessenkonflikte bestehen.
